# High Throughput *In vivo* Analysis of Plant Leaf Chemical Properties Using Hyperspectral Imaging

**DOI:** 10.3389/fpls.2017.01348

**Published:** 2017-08-03

**Authors:** Piyush Pandey, Yufeng Ge, Vincent Stoerger, James C. Schnable

**Affiliations:** ^1^Department of Biological Systems Engineering, University of Nebraska-Lincoln Lincoln, NE, United States; ^2^Agricultural Research Division, University of Nebraska-Lincoln Lincoln, NE, United States; ^3^Center for Plant Science Innovation, University of Nebraska-Lincoln Lincoln, NE, United States; ^4^Department of Agronomy and Horticulture, University of Nebraska-Lincoln Lincoln, NE, United States

**Keywords:** high throughput plant phenotyping, hyperspectral imaging, water content, macronutrients, micronutrients, chemical sensing

## Abstract

Image-based high-throughput plant phenotyping in greenhouse has the potential to relieve the bottleneck currently presented by phenotypic scoring which limits the throughput of gene discovery and crop improvement efforts. Numerous studies have employed automated RGB imaging to characterize biomass and growth of agronomically important crops. The objective of this study was to investigate the utility of hyperspectral imaging for quantifying chemical properties of maize and soybean plants *in vivo*. These properties included leaf water content, as well as concentrations of macronutrients nitrogen (N), phosphorus (P), potassium (K), magnesium (Mg), calcium (Ca), and sulfur (S), and micronutrients sodium (Na), iron (Fe), manganese (Mn), boron (B), copper (Cu), and zinc (Zn). Hyperspectral images were collected from 60 maize and 60 soybean plants, each subjected to varying levels of either water deficit or nutrient limitation stress with the goal of creating a wide range of variation in the chemical properties of plant leaves. Plants were imaged on an automated conveyor belt system using a hyperspectral imager with a spectral range from 550 to 1,700 nm. Images were processed to extract reflectance spectrum from each plant and partial least squares regression models were developed to correlate spectral data with chemical data. Among all the chemical properties investigated, water content was predicted with the highest accuracy [*R*^2^ = 0.93 and RPD (Ratio of Performance to Deviation) = 3.8]. All macronutrients were also quantified satisfactorily (*R*^2^ from 0.69 to 0.92, RPD from 1.62 to 3.62), with N predicted best followed by P, K, and S. The micronutrients group showed lower prediction accuracy (*R*^2^ from 0.19 to 0.86, RPD from 1.09 to 2.69) than the macronutrient groups. Cu and Zn were best predicted, followed by Fe and Mn. Na and B were the only two properties that hyperspectral imaging was not able to quantify satisfactorily (*R*^2^ < 0.3 and RPD < 1.2). This study suggested the potential usefulness of hyperspectral imaging as a high-throughput phenotyping technology for plant chemical traits. Future research is needed to test the method more thoroughly by designing experiments to vary plant nutrients individually and cover more plant species, genotypes, and growth stages.

## Introduction

High throughput plant phenotyping based on analysis of image data has recently emerged as a new frontier field for plant breeding and crop improvement. Modern plant breeding and crop improvement efforts depend on a combination of genotypic and phenotypic data. Advances in sequencing technology have drastically reduced the time span, labor, and cost required to generate genotypic data. However, the collection of plant phenotypic data has proven more recalcitrant to increases in throughput and/or decreases in cost, and now represents the rate-limiting step in plant breeding efforts (Houle et al., 2010; Furbank and Tester, 2011; Fiorani and Schurr, 2013). The goal of high throughput plant phenotyping technologies is to enable the collection of large-scale plant morphological and physiological traits rapidly and non-destructively. Image processing pipelines have been developed to extract image-based trait measurements that are not possible with the conventional destructive methods (Klukas et al., 2014; Knecht et al., 2016). A majority of early research on high throughput plant phenotyping largely focused on the model plant *Arabidopsis thaliana*, but it has quickly expanded to a number of important field crops including wheat (Golzarian et al., 2011), barley (Chen et al., 2014), rice (Campbell et al., 2015), foxtail millet (Fahlgren et al., 2015a), sorghum (Neilson et al., 2015), and maize (Ge et al., 2016). These studies demonstrated the usefulness and benefit of image-based high throughput phenotyping for characterizing plant growth, biomass and yield, and the temporal dynamics of changes in these traits across different stages of development.

RGB (Red Green Blue) images capture data on light intensities in three spectral bands corresponding to the responses of the three types of cones in the human eye. RGB images are collected using the same imaging technologies widely employed in consumer cameras. As a result, these sensors usually have the lowest cost and the highest pixel resolution. RGB image data is most commonly used to measure plant size, biomass, and growth rate. Fluorescence imaging quantifies fluorescence intensity of excited chlorophyll molecules in Photosystem II, and is used to measure photosynthetic activities of plants (Jansen et al., 2009). Thermal infrared (TIR) imaging measures the temperature of the plant leaf surface, and therefore has the potential to quantify stomata resistance and water evaporation from the leaves (Sirault et al., 2009). Furthermore, 3D imaging systems are also investigated to quantify the 3D structure of the plants (Chéné et al., 2012). There are two clear advantages of image-based phenotyping. Firstly, plants can be subject to several of these imaging modalities, allowing non-destructive evaluation of many aspects of plant traits simultaneously. Secondly, imaging by its nature is non-destructive, allowing trait measurements at multiple time points along plants' life cycle which enables temporal dynamic analysis.

Non-imaging spectroscopy can quantify light intensity across hundreds or thousands of distinct spectral bands but does not provide data on spatial variation. These bands are commonly divided into the visible (VIS, 400–700 nm), near infrared (NIR, 700–1,100 nm) and short wave infrared (SWIR, 1,100–2,500 nm) spectral regions. Non-imaging spectroscopy is commonly used as a remote, non-destructive method for rapid analysis of many properties for both fresh leaves and processed plant materials. VIS is the region where photosynthetic pigments such as chlorophylls, carotenoids, and xanthophyll absorb strongly; whereas reflection in NIR is dominated by structural reflection of turgid plant cells. These two regions combined are often employed to probe the properties of living plant leaves such as pigment concentrations, water content, and light use efficiencies (Penuelas and Filella, 1998; Sims and Gamon, 2002; Blackburn, 2007). Many empirical and semi-empirical spectral indices—such as Normalized Difference Vegetation Index (NDVI), Photochemical Reflectance Index (PRI), and chlorophyll index—have been designed and derived from the leaf reflectance spectra and found to be correlated with plant physiological status. Biological samples are abundant in chemical bonds that absorb strongly in the mid infrared region, and those strong vibrational bands cause discernible bands (their combinations, 1st overtone and 2nd overtone) in the SWIR region. This forms the chemical basis of using SWIR to quantify properties such as protein, phosphorus, cellulose, hemicellulose, and mineral (ash) contents in the processed (usually dried and ground) plant materials (Batten, 1998; Curran et al., 2001). Multivariate modeling techniques such as principal component regression and partial least squares regression are often used in these applications to model the association between plant chemical data and spectral data.

Due to these successful uses of non-imaging spectroscopy on plant characterization, hyperspectral imaging (or imaging spectroscopy) is widely recognized as an imaging modality of great potential for high throughput plant phenotyping (Li et al., 2014; Fahlgren et al., 2015b). Hyperspectral imaging combines features of both RGB imaging (multiple distinct pixels) and spectroscopy (intensity data from many different distinct spectral bands). It has the potential to complement other imaging modules by enabling the measurement of plant chemical traits (such as water, nitrogen, and nutrient levels), rendering a more complete characterization of plant traits. Because hyperspectral imaging measures the entire spectrum at each plant pixel, versatile analytical techniques (such as multivariate statistical modeling) can be employed, which could further expand its utility in phenotype formulation and extraction.

Several authors have used hyperspectral imaging to characterize the spectral variations of field crops under drought stress (Römer et al., 2012) or disease stress (Mahlein et al., 2012; Bauriegel and Herppich, 2014). Field phenotyping systems that incorporate hyperspectral imaging are also reported (Busemeyer et al., 2013; Virlet et al., 2016). Nevertheless, the literature is scant in the use of hyperspectral imaging to quantitatively measure chemical properties of plants at the single plant level (i.e., greenhouse phenotyping). In this study, we conducted experiments in a high throughput phenotyping greenhouse equipped with a hyperspectral imaging system to investigate how effectively hyperspectral imaging can quantify plant chemical properties including water content and macronutrient and micronutrient concentrations in plant leaves.

## Materials and methods

### Hyperspectral camera and imaging chamber

**Figure 1:**
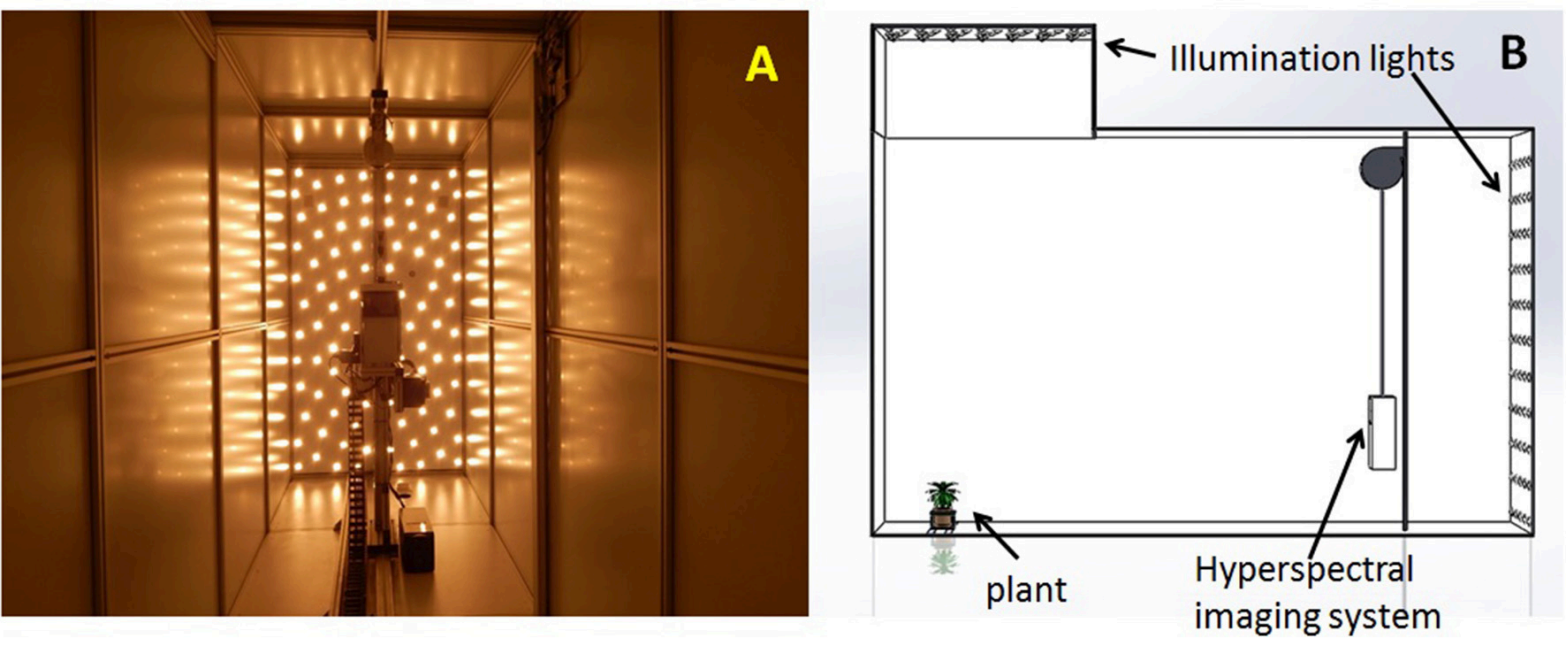
**(A)** The interior view of the hyperspectral imaging chamber. **(B)** The 3D rendering showing the setup of the imaging chamber.

The hyperspectral imaging system is the part of high throughput phenotyping greenhouse (Scanalyzer3D, LemnaTec GmbH, Aachen, Germany) installed at the University of Nebraska-Lincoln. The hyperspectral camera is an extended VNIR (visible and near infrared), push-broom type imaging spectrometer (Headwall Photonics, Fitchburg, MA, USA). Both plants and the camera remain still during image acquisition. A scanning mirror inside the imaging system is rotated to sequentially expose each line of image from the top to the bottom of the imaging chamber. The camera has a nominal spectral range from 550 to 1,700 nm (the green-red portion of VIS, the entire NIR, and the first part of SWIR). The spectral interval of each image band is 4.77 nm, giving a total of 243 image bands for each hyperspectral image cube. In the spatial domain, the imaging detector array is 320-pixel wide. The mirror's incremental angle at each step is configured such that the pixel size at the scanning (vertical) direction is 5 mm. Each hyperspectral image is consisted of 500 scan lines, which covers the height of the imaging chamber (2,500 mm = 5 × 500). The imaging system is placed in the imaging chamber such that the scan line matches the entire width of the imaging chamber (1,600 mm). Therefore, the image pixel size on the horizontal direction is also 5 mm (1,600/320, meaning square pixels). The imaging chamber is illuminated by two banks of halogen lamps (35W, color temperature 2,600 K), one at the back of the imaging chamber behind the hyperspectral imaging system and the other on the top. The interior view of the hyperspectral imaging chamber during image acquisition is shown in Figure 1, as well as a 3D rendering of the chamber showing its relative dimension. To reiterate the setup of the hyperspectral system: 500 pixels vertical (5 mm per pixel) × 320 pixels horizontal (5 mm per pixel) × 243 spectral bands (4.7 nm wide from 550 to 1,700 nm).

### Experiment design and data collection

Sixty maize (Zea mays, B73 inbred) plants and 60 soybean (Glycine max, Thorne variety) plants were used in this study. Seeds were germinated and grown in 5.7 L pots having a diameter of 22 cm and a height of 20 cm filled with germination mix (Fafard). The temperature in the greenhouse was maintained between 25 and 27°C during daytime and 20–22°C during the night time. Relative humidity was maintained at ~60%. The daily light intensity resulting from natural sunlight and the supplemental LED peaked at ~350 μmol/m2/s photosynthetically active radiation. The supplemental LED had a lighting period set to 12 h (7:00 to 19:00 h). Plant growth and data collection occurred in June and July of 2016.

The maize and soybean plants were divided equally into two different experiments. The first was a water limitation experiment including 30 maize and 30 soybean plants. The purpose of this experiment was to create a wide range of plant leaf water contents for the testing of hyperspectral imaging for plant water content measurement. These 60 plants were further divided into two groups: a control group and a water limitation group. For the control group, water was added daily to each pot to field capacity (water potential −33 kilo Pascal). For the water limitation group, water was added daily to each pot to field capacity until 20 Days after Sowing (DAS) for maize and 27 DAS for soybean. This was to ensure that non-stressed plants were established before they were subject to water limitation. After that, only limited amount of water was added to soybean plants (to prevent complete dry out) whereas no water was given to the maize plants.

The remaining 30 maize and 30 soybean plants were used in the second nutrient stress experiment. These maize plants were divided into groups of 10 plants each and were subjected to three different nutrient levels: low, medium, and high. The same procedure was also applied to the soybean plants. The purpose was to create a large range of nutrient levels (in particular nitrogen, phosphorus, and potassium) in plant leaves for the testing of the hyperspectral imaging system. The treatments were administered using a slow release commercial fertilizer (Osmocote Pro 5-6 mo. 17-5-11 with improved micronutrients). The application rate was 20 and 7.5 g per kg of soil for high and medium levels, respectively; while no fertilizer was applied to the low nutrient group.

Data collection was done between 27 and 64 DAS, during which the maize and soybean plants spanned several developmental stages. The plants under water limitation were sampled earlier (until 51 DAS) so that the plants under severe water limitation can be sampled before complete dry out. Most of the plants in the nutrient stress experiment were therefore sampled toward the end of the experiment. This was beneficial since it allowed these plants to grow to larger total biomass and measurable nutrient deficiency was developed for the plants receiving medium or low nutrients.

Data collection and imaging was conducted on 10 different days. On each day, 12 plants were selected. The plants were placed on Scanalyzer3D's conveyor belt and transported to system's imaging chamber where the hyperspectral images were acquired. Immediately following each plant, an empty pot carrier was also sent to the imaging chamber and a blank image was taken as the reference image. This reference image was used to normalize the raw digital numbers in plant images to a common range between 0 and 1. This normalization step helped to account for the short-term and long-term variations in halogen lamps' energy output and imaging detector responses.

After image acquisition, the plants were cut above the soil line to determine the fresh weight (W_fresh_) of plant shoot. The harvested plant shoot materials were placed in a walk-in drying oven at 50°C for over 72 h to obtain dry weight (W_dry_). The actual drying time was determined by repeatedly weighing the dry plant material every 24 h until two consecutive measurements were found to be equal. Plant water content was calculated WC = (W_fresh_ − W_dry_)/W_fresh_ × 100%. The dried plant leaves were sent to a commercial lab (Midwest Laboratories, Omaha, NE) where the samples were ground, homogenized, and analyzed to determine the concentration of macronutrients including total nitrogen (N), phosphorus (P), potassium (K), magnesium (Mg), calcium (Ca), and sulfur (S), and micronutrients including sodium (Na), iron (Fe), manganese (Mn), boron (B), copper (Cu), and zinc (Zn). N was analyzed by Dumas method with a LECO FP428 nitrogen analyzer (AOAC method 968.06). All other elements were analyzed with microwave nitric acid digestion followed by inductively coupled plasma spectroscopy (AOAC method 985.01).

### Hyperspectral image analysis and data analysis

**Figure 2:**
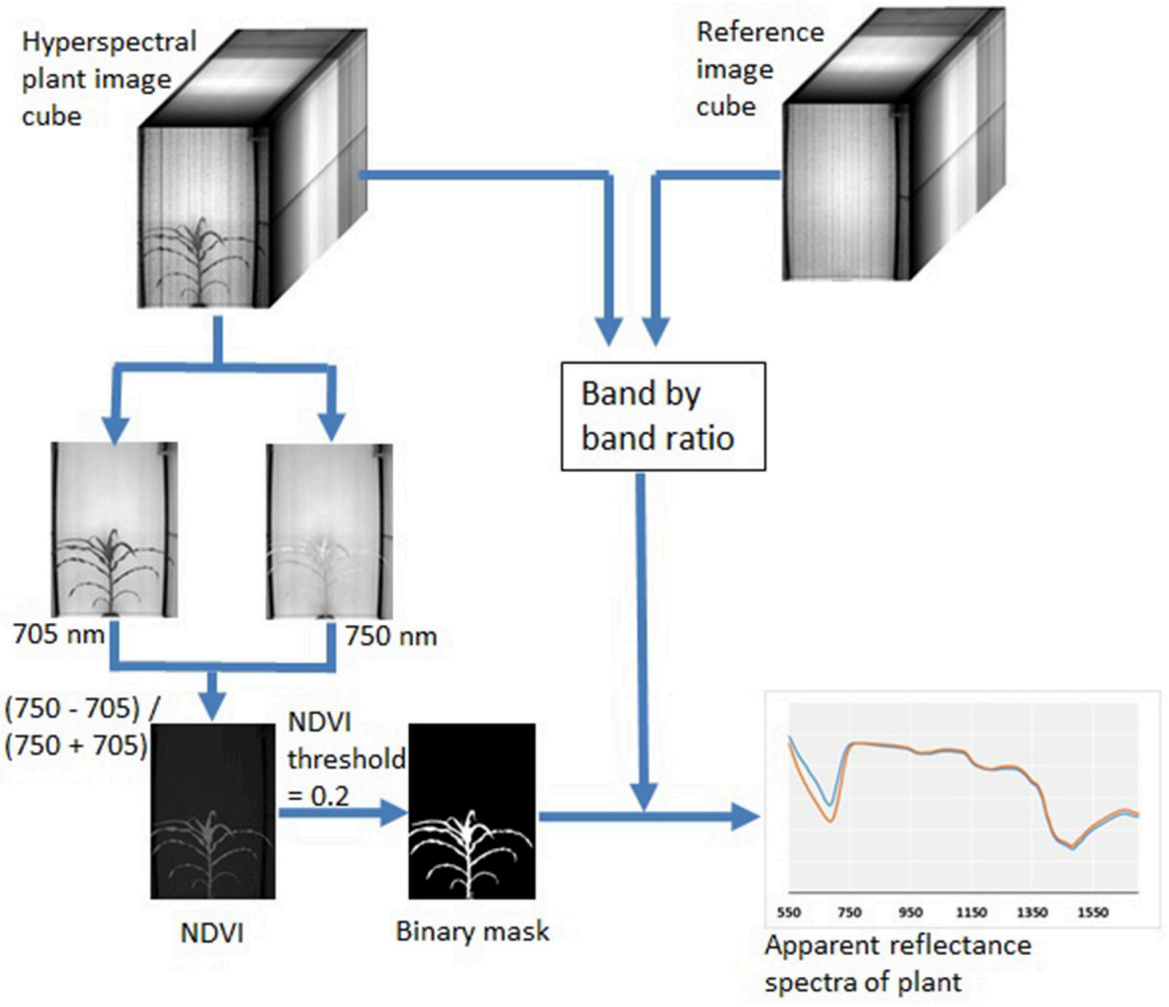
Procedures for hyperspectral plant image analysis to obtain apparent reflectance spectra.

Raw hyperspectral image data were in 16 bit BIL (Band Interleaved by Line) format. A customized function was written to convert both the raw image data (including both 120 plant images and 120 reference images) into 3D image cubes. The plant image cubes were then divided by the reference image cubes through a band-by-band operation, which converted plant image pixels from the raw intensity numbers to a fractional number (between 0 and 1) analog to leaf reflectance. Two image bands at 705 and 750 nm (I_705_ and I_750_) were extracted from the image cubes to calculate an NDVI image as (I_750_−I_705_)/(I_750_+I_705_). This NDVI image was found very effective to segment plants from the background by setting a universal threshold of 0.20. After segmentation, the binary image was used to extract plant pixels from all image bands along the wavelength. Extracted plant pixels from each image band were then averaged to obtain the average reflectance of the plants. Through these steps, the hyperspectral image was reduced to a 243 data-point spectral curve that represented the average apparent reflectance of each plant from 550 to 1,700 nm. Figure 2 summarizes the image processing procedure and the example hyperspectral images from this study. Image processing was implemented in MATLAB (version 2015b, Mathwork®, Natick, MA) with the Image Processing Toolbox.

Principal component analysis was first implemented on spectral data to detect possible outliers in the spectral space. Partial least squares regression (PLSR) was used to model and predict water content, macronutrient, and micronutrient concentrations for maize and soybean plants from their reflectance spectra. We split the 120 plants into two groups: 60 plants (50%) for model calibration and the other 60 (50%) for independent model validation. The split was done such that the species (maize vs. soybean) and the treatment levels for both water stress and nutrient stress experiments were equally presented in the calibration and validation set. A check was done to ensure there was no significant difference in the response variables between the two sets (Supplementary Table 1), which was important for effective modeling. For model calibration, the scheme of leave-one-out cross validation was employed. Models having as many as 12 latent factors were considered, and the size of the models was determined by choosing the number of factors that gave the minimum root mean squared error of cross validation (RMSE_CV_). The following statistics were calculated for model evaluation: Coefficient of Determination (*R*^2^) between lab-measured and model predicted values, RMSE (Equation 1), Ratio of Performance to Deviation (RPD, Equation 2), and Mean Absolute Percent Error (MAPE, Equation 3). These statistics were calculated for both cross validation (in model calibration) and validation. PLSR modeling was performed in R statistical environment (R Core Team, 2016) with “chemometrics” package.

(1) $$\begin{array}{c} RMSE=\sqrt{\frac{1}{N}\times \sum^{\text{​}}{\left(y_{i}-{\hat{y}}_{i}\right)}^{2}} \end{array}$$

(2) $$\begin{array}{c} RPD=SD/RMSE \end{array}$$

(3) $$\begin{array}{c} MAPE=\frac{\frac{1}{N}\times \sum^{\text{​}}|y_{i}-{\hat{y}}_{i}|}{mean}\times 100\% \end{array}$$

N is the number of plants in the calibration or validation set (60); y_*i*_ and ŷ_*i*_ are the lab-measured and model-predicted values, respectively; SD and mean are the standard deviation and mean of the lab-measured values.

## Results

### Effects of water and nutrient treatments on plant chemical concentrations

**Figure 3:**
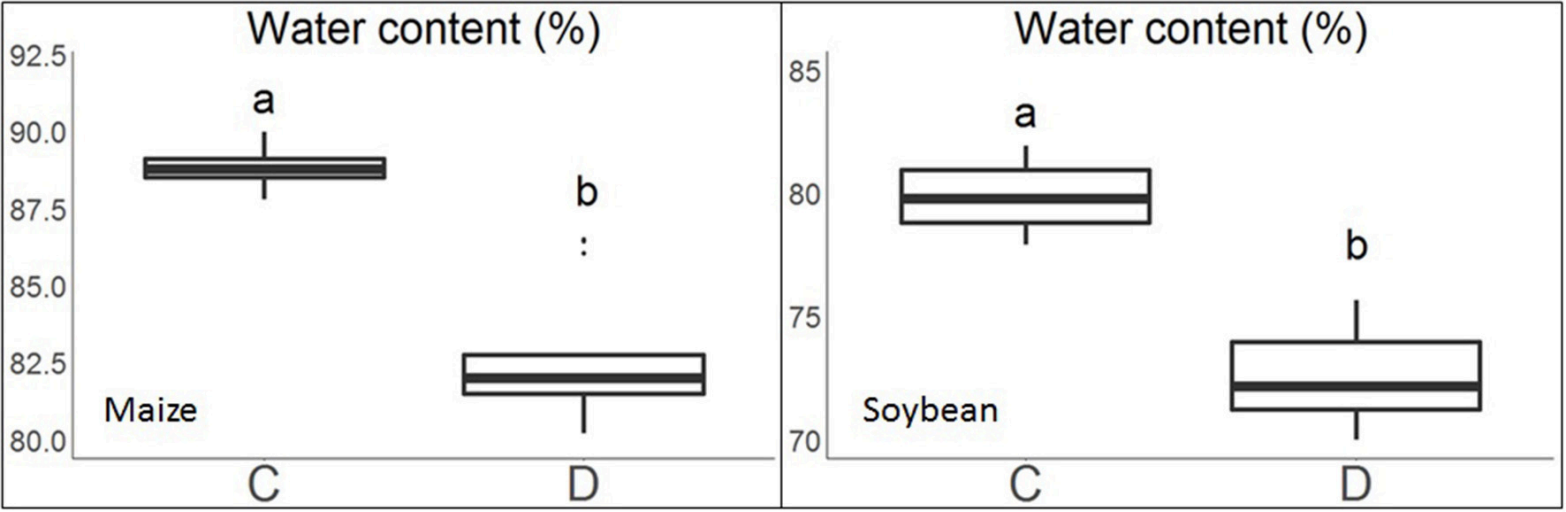
Boxplots of leaf water content of the maize and soybean plants under the control (C) and water limitation (D) treatments. Treatment groups assigned to different letters indicate their means were significantly different with Tukey's Honest Distance test (*p* < 0.05).

The experimental conditions described above created large variation in plant water content across water treatments. Plant water content ranged from 79.6 to 91.0% for maize and 68.2 to 81.9% for soybean (Figure 3). It can also be seen that plants under water limitation had significantly lower leaf water content than those under control.

**Figure 4:**
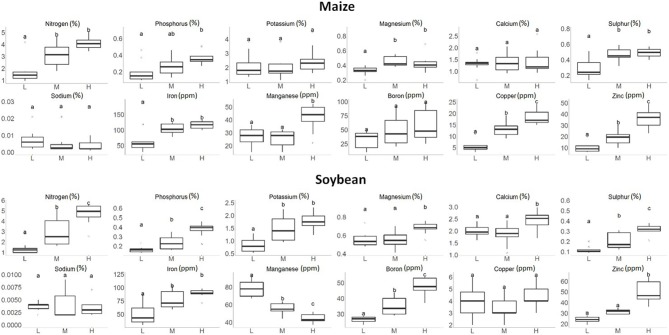
Boxplots of the macronutrient and micronutrient concentrations in maize and soybean plant leaves under the low (L), medium (M), and high (H) nutrient treatments. Treatment groups assigned to different letters indicate their means were significantly different with Tukey's Honest Distance test (*p* < 0.05).

Figure 4 shows the range of macronutrient and micronutrient levels observed in maize and soybean leaves under the high, medium, and low nutrient application rates. It can be seen that these traits responded to the nutrient treatment quite differently. For most nutrients, plants grown with higher nutrient rates tended to have higher concentrations of both macronutrients and micronutrients in leaves; even though in several cases, there were no statistically significant differences among all three nutrient application rates (such as Na for either maize or soybean). As mentioned above, our goal was to create wide ranges in macronutrients and micronutrients in plant leaf tissues, and Figure 4 indicates that this goal was successfully achieved for the majority of target nutrients. For example, the N concentration in maize and soybean plants combined ranged from 0.96 to 5.68%. This range was wider than what would be found for maize or soybean under agronomically relevant field conditions, and would benefit PLSR model calibration with hyperspectral imaging.

### PLSR modeling of plant leaf chemical traits

**Figure 5:**
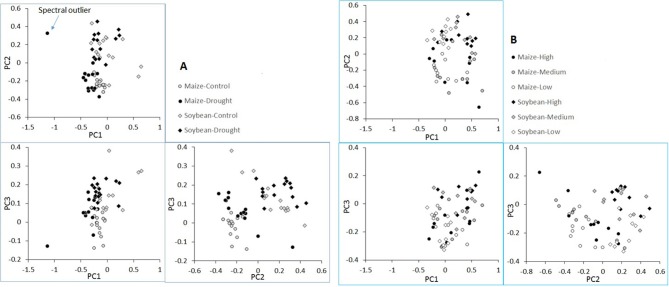
Pairwise score plots of the first three principal components of plant spectra from the water limitation experiment **(A)** and the nutrient stress experiment **(B)**.

Pairwise score plot of the first three principal components (PC1, PC2, and PC3) of the plant spectra from the water limitation experiment is shown in Figure 5A. The plot revealed one potential outlier in the spectral space. Further examination of this maize plant, however, indicated that its spectral and chemical data did not deviate substantially from other samples. This sample was therefore retained in the subsequent analysis. The plot also revealed that maize and soybean plants were largely separated from each other (in PC1 vs. PC2 and PC2 vs. PC3 plots). In addition, plants receiving the different water treatments was also separable (in PC1 vs. PC3 plot), although separation was not as pronounced as plant species. Similarly, Figure 5B shows the pairwise PC plot of plant spectra from the nutrient stress experiment. Still maize and soybean plants were largely separable (again PC1 vs. PC2 and PC2 vs. PC3 plots). However, different from the water treatments, no clear spectral separation was seen among the nutrient stress levels. Overall, it suggested that plant species contributed most to spectral differences, followed by the water treatments and then the nutrient treatments. This had important implications for PLSR modeling, which is discussed later with the results of PLSR modeling.

The results of PLSR analysis (Table 1) show that, among all the plant chemical traits studied, leaf water content can be modeled with the highest accuracy (*R*^2^ = 0.93 and MAPE = 1.6%) for validation. For macronutrients, N, P, K and S were modeled quite satisfactorily, with their validation *R*^2^ > 0.80 and MAPE < 15%. Mg and Ca were modeled with moderate accuracy, with their validation *R*^2^ around 0.70 and MAPE around 15%. Compared to macronutrients, micronutrients were in general modeled with lower accuracy. Cu and Zn were modeled most successfully in this group, with validation *R*^2^ of 0.86 and 0.73 (MAPE of 20.8 and 16.1%), respectively. Modeling of Fe and Mn showed somewhat lower accuracy, with validation *R*^2^ slightly greater than 0.60 (MAPE of 13.7 and 17.3%). Models of Na and B showed the lowest prediction accuracy among all variables tested, with validation *R*^2^ lower than 0.30. MAPE of Na was 49.5%, much higher than all other variables.

**Table 1:** Cross-validation and validation results of using hyperspectral images to predict plant leaf water content, macronutrients, and micronutrient concentrations with partial least squares regression.

	**Cross-validation (*****n*** = **60)**					**Validation (*****n*** = **60)**			
	***R*^2^**	**RMSE_CV_**	**RPD**	**MAPE (%)**	**Model size**	***R*^2^**	**RMSE_V_**	**RPD**	**MAPE (%)**
WC (%)	0.97	1.18	5.64	1.1	12	0.93	1.62	3.80	1.6
N (%)	0.88	0.47	2.94	8.8	12	0.92	0.41	3.60	8.3
P (%)	0.71	0.075	1.86	13.8	10	0.83	0.056	2.43	12.3
K (%)	0.73	0.53	1.92	15.5	7	0.83	0.41	2.47	14.1
Mg (%)	0.69	0.088	1.81	13.1	5	0.69	0.078	1.79	12.2
Ca (%)	0.75	0.35	2.02	14.6	8	0.70	0.39	1.62	15.7
S (%)	0.71	0.068	1.88	13.0	11	0.83	0.052	2.46	12.2
Na (%)	0.19	0.003	1.13	46.2	7	0.18	0.003	1.09	49.5
Fe (ppm)	0.73	16.0	1.95	10.4	11	0.68	16.2	1.70	13.7
Mn (ppm)	0.51	11.1	1.45	21.2	7	0.64	9.56	1.62	17.3
B (ppm)	0.38	10.4	1.29	20.2	7	0.29	15.6	1.12	23.3
Cu (ppm)	0.80	3.01	2.25	24.9	12	0.86	2.52	2.69	20.8
Zn (ppm)	0.64	7.02	1.68	15.3	8	0.73	7.39	1.93	16.1

In analytical chemistry and chemometric modeling, RPD is widely used as a criterion to evaluate the usefulness of prediction models by normalizing model's RMSE with the standard deviation of the reference value. Models with an RPD > 2.0 are generally considered good models, with RPD > 3.0 indicative of quantitative analysis. Models with RPD between 1.5 and 2.0 are considered fair in a quantitative sense, but they can be useful for qualitative screening and have potential for improvement (Chang et al., 2001; Fearn, 2002). The majority of the models developed in this study had RPD between 1.5 and 4.0 (except for Na and B). Therefore, these models would be useful for the screening purpose of leaf biochemical traits. In many circumstances of plant breeding and phenotypic scoring, it is not necessary to precisely predict traits. Rather, the goal often is to rapidly screen out the superior lines from a population of hundreds or even thousands of lines. In this sense, the models developed from hyperspectral imaging in this study can be used as a practical tool for rapid screening of plant leaf biochemical traits.

## Discussion

The result that water content can be predicted from hyperspectral imaging is not surprising. Water is a strong absorber in the NIR and SWIR spectral range, and the variation of reflected light energy is therefore sensitive to probe water status in plants. In fact, spectral indices have been constructed from known water absorption bands (such as those at 970, 1,240, 1,450 nm) as proxies for plant canopy water content in remote sensing (Gao, 1996; Penuelas et al., 1997).

As an alternative to hyperspectral imaging, researchers have tested the use of a single band NIR camera (with a wide spectral response covering 900–1,700 nm) to quantify plant water content (Chen et al., 2014; Neilson et al., 2015). Although, NIR images are much easier to process, their studies were only able to establish a qualitative relationship between the single band NIR images and leaf water content. This study suggests that hyperspectral imaging provides substantially more accurate predictions of leaf water content. The improved accuracy of predictions with hyperspectral imaging relative to broad spectrum NIR imaging can be attributed to two factors. Firstly, hyperspectral imaging captures reflectance at many contiguous narrow spectral bands, allowing the water absorption bands to be better resolved. Secondly, PLSR modeling (which is not possible with single band NIR images) is highly efficient in extracting useful spectral information correlated with water content even in the presence of confounding factors and noises. This result also confirms the conclusions of a previous study we conducted that showed hyperspectral imaging predicted plant leaf water content across two contrasting maize genotypes successfully (Ge et al., 2016).

The fact that N, P, and S can be modeled satisfactorily with hyperspectral imaging was also anticipated. As mentioned in the introduction section, these elements usually participate in covalent bonding of carbon compounds that usually absorb VIS-NIR-SWIR light energy and lead to their quantification. For example, N in plant leaves exists in several forms including proteins, free amino acids, and within chlorophyll molecules. Protein and free amino acids contain N-H bonds that absorb in SWIR region, whereas chlorophylls are pigments with strong absorption in the VIS region. The same is true for P (such as sugar-phosphate intermediates and phospholipids) and S (amino acids), but with lower concentrations in living plant tissues than N.

Macronutrients K, Ca, Mg, and Na and micronutrients Fe, Mn, Zn, Cu are all metallic elements and they exist primarily as ions in living plant tissues. In ionic form, they do not produce active spectral absorption features in the VIS-NIR-SWIR region. It is noteworthy that some of these ions do bond electrostatically or as ligands to larger carbon containing compounds, which give spectroscopic basis for their quantification with hyperspectral imaging. For example, Mg is a part of the ring structure of chlorophyll molecules (which is a strong absorber in VIS); whereas Fe is associated with cytochrome involved in the electron transfer of photosynthesis. Furthermore, deficiency in these macronutrients and micronutrients usually cause distinct visual symptoms (such as chlorosis and necrosis of leaves and veins) in plants, which can be readily captured by hyperspectral imaging.

**Figure 6:**
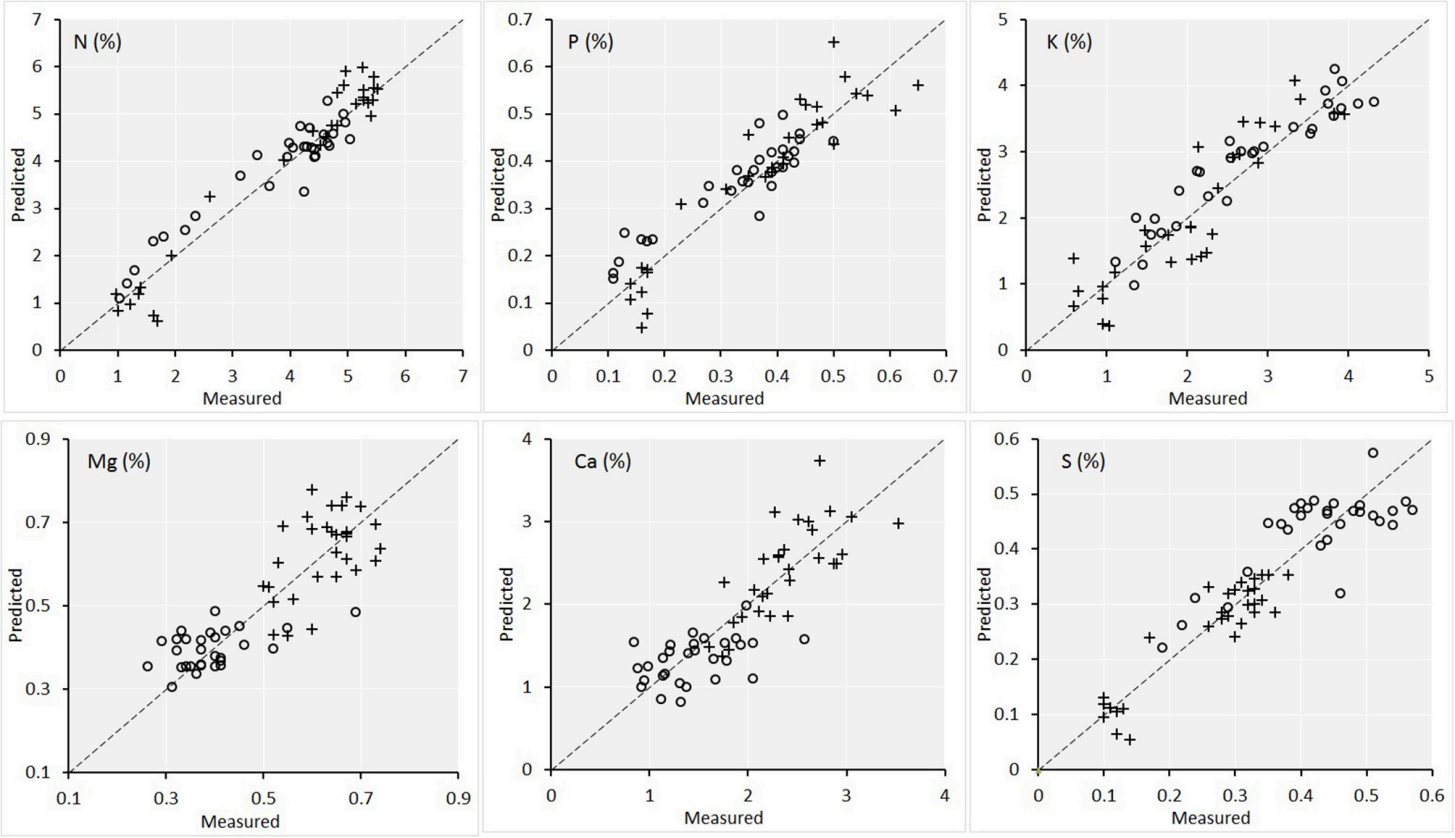
Scatterplot of the lab measured value vs. the image predicted value of the concentrations of the six macronutrients in plant leaves for the validation set (*n* = 60). Maize plants are denoted by circles and soybean plants are denoted by crosses. The statistics of the plots are given in Table 1.

**Figure 7:**
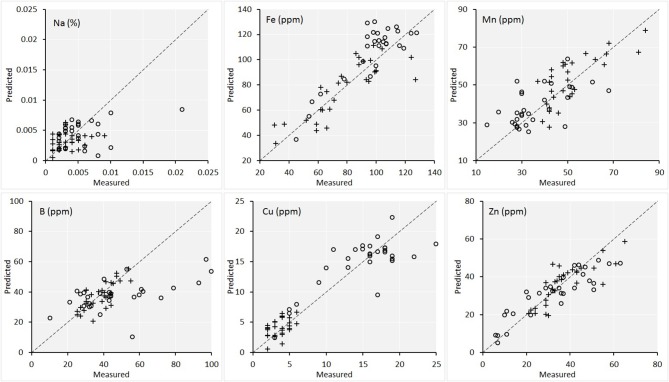
Scatterplot of the lab measured value vs. the image predicted value of the concentrations of the six micronutrients in plant leaves for the validation set (*n* = 60). Maize plants are denoted by circles and soybean plants are denoted by crosses. The statistics of the plots are given in Table 1.

Figures 6, 7 are the scatterplots showing the relationships of lab-measured and image-predicted plant macronutrient and micronutrient concentrations. These plots provided additional insight into how the PLSR predictions were made between maize and soybean. Firstly, the scatter of maize and soybean plants around 1:1 line were quite consistent. In other words, no systematic overestimation or underestimation were observed for either maize or soybean. This is the desirable attribute of PLSR modeling. Because the calibration set included both maize and soybean plants, the PLSR models calibrated were able to predict the plant traits on both crops.

Secondly, for the traits including Mg, Ca, and Cu, there were natural separations among maize and soybean. Because Figure 5 also showed a large separation of spectra among the two species, the concern is that PLSR models captured the between-species variation successfully but might not perform well within each species. To shed more light on this point, we conducted PLSR modeling involving only one species for these three traits (with leave-one-out cross validation). The results were as follows. For Maize-Mg model, *R*^2^ = 0.64 and RPD = 1.75; for Soybean-Mg model, *R*^2^ = 0.69 and RPD = 1.83; for Maize-Ca model, *R*^2^ = 0.67 and RPD = 1.68; for Soybean-Ca model, *R*^2^ = 0.70 and RPD = 1.80; for Maize-Cu model, *R*^2^ = 0.75 and RPD = 1.92; for Soybean-Cu model, *R*^2^ = 0.65 and RPD = 1.63. Therefore, the performances of these within species models were comparable to those of the models that included both species (Table 1). Supplementary Table 2 gives the full cross validation results (*R*^2^ and RPD) of all 13 variables by modeling maize and soybean plants separately, for interested readers.

With RGB imaging, simple linear regressions are commonly used to relate images (such as green pixel count) to plant traits (such as biomass). However, hyperspectral imaging presents different challenges and opportunities. Analyzing hyperspectral data requires more complicated statistical modeling to relate measured reflectance to plant chemical traits. In this study, we employed PLSR, but many other multivariate statistical modeling techniques such as random forest (Baranowski et al., 2015), support vector machines (Behmann et al., 2014), and artificial neural networks (Singh et al., 2016) can also be used. This can be regarded as an advantage of hyperspectral imaging, because these more sophisticated (and non-linear) modeling techniques could potentially improve the prediction accuracy. One potential problem with the use of these advanced modeling tools is that, as pure data driven approaches, the models might be difficult to interpret for their biological significance. PLSR models for the selected nutrients are given in Supplementary Figure 1 for interested readers.

Although the results are promising, several limitations of our study should also be pointed out. Firstly, we used a commercial slow release fertilizer for the nutrient treatment. While this created desirable variations in all macro and micronutrients, it did not target for the variation of any specific nutrient in a clearly defined way. The concern is that deficiency of multiple nutrients and their interdependence could lead to false correlations between the nutrients and spectral data. Pairwise correlation analysis (Supplementary Figure 2) indicated some level of covariation among the nutrients. Therefore, further studies are needed to vary nutrient concentration individually and test the efficacy of hyperspectral imaging under those conditions. This would establish the validity of this method for plant chemical sensing and as a tool to help address questions of biological relevance.

Secondly, the models developed in this study involved only two crops (maize and soybean), each represented by only a single accession. A plant breeding or plant genetics focused high throughput phenotyping project will generally involve dozens to hundreds of genotypes which may exhibit other types of compositional variation creating more noise in the analysis. Another limitation of the analysis conducted in this study was that imaging only took at certain developmental stages (for maize V8 to V14 and for soybean V5 to before flowering). To fully utilize hyperspectral imaging for routine high throughput phenotyping, further studies should be undertaken to understand how models relating plant reflectance to chemical properties will be affected by (1) plant species, (2) different genotypes within a species, and (3) plant's developmental stages. It may prove to be the case that overall models can be developed to make satisfactory prediction. But much more likely, plant specific or genotype specific or developmental stage specific models will be needed for satisfactory prediction of these chemical traits under different experimental conditions and scenarios.

### Practical considerations to use hyperspectral imaging for high throughput plant phenotyping

Hyperspectral imaging is significantly different from some other imaging modalities such as RGB and thermal infrared (TIR) imaging. RGB is most widely used to extract green plant pixels which in turn are counted to estimate plant biomass. In this process, the intensities of the R G B components of plant pixels are not necessarily of interest to the users, as long as those plant pixels can be successfully segmented from background pixels. TIR imaging involves the detection of emitted electromagnetic energy (at much longer wavelengths of 8–14 μm) from plant leaves and therefore no illumination is needed. For these reasons, effort was not usually emphasized to radiometrically calibrate the RGB and TIR cameras.

On the other hand, the use of hyperspectral imaging to predict chemical properties of plants relies on the accurate measurement of reflectance of plant pixels at each waveband. This means that radiometric calibration is a key step in the collection of informative hyperspectral images. Radiometric calibration seeks to account for the short- and long-term variations in the spectral output intensity of light sources (halogen lamps) and imaging detectors' response and dark current. Ideally, a calibration after each plant measurement should be implemented to obtain the highest performance. This level of calibration has been implemented for hyperspectral imaging applications in some other fields including quality assessment of food products (Naganathan et al., 2008) and fruits and vegetables (Qin and Lu, 2008). However, more frequent calibrations create a tradeoff between image quality and analysis throughput, which are two important factors to consider in high throughput phenotyping.

Whereas, image acquisition by RGB is almost instantaneous, the time needed for hyperspectral image acquisition is substantially longer. With our system, acquiring one hyperspectral image cube takes ~2 min (due to line-by-line scanning mechanism of image formation). If a reference image is also acquired each time a plant is imaged, 4–5 min are therefore needed to scan one plant. This represents a significant rate limiting factor for high throughput phenotyping. With this speed, a work shift of 12 h continuous imaging can only scan about 180 plants. This is obviously not adequate for many large population studies which usually involve several hundreds of plants. Therefore, from an operational and logistics standpoint, system characterization is needed so that the researchers would know how often a calibration should be run to strike a balance between radiometric accuracy and imaging throughput.

### Whole plant vs. multiple predictions per plant

In this study, we treated the plants as a whole. All leaves from the plant were harvested, dried and thoroughly homogenized and a portion of it was sent to the lab for analysis. At the image analysis side, all pixels of the plant were combined to obtain an average reflectance at each wavelength. Therefore, the prediction models developed in our study specified the correlations between spectra and the chemical traits of plants at the whole plant level (i.e., one prediction per plant per image time). However, it is possible to make spatially resolved predictions for the plant, because a complete spectrum can be extracted from every pixel of the plant. This would provide a tool to quantify the spatial distribution of these chemical properties within the plant. If such quantifications could be made at multiple time points along plants' life cycle, it would be possible to elucidate (1) how the macronutrient and micronutrient elements are taken up from the soils, and (2) the translocation of mobile nutrients (such as N, P, K) among plant tissues as plants develop. Currently, there is significant variation in image quality throughout the chamber as a result of non-uniform lighting (even after normalization), which makes accurate pixel by pixel prediction challenging. However, this is a solvable problem; and, in the future it should be possible to improve the lighting uniformity inside the chamber and retrieve the true reflectance of plant leaves from each plant pixel, which would enable spatially resolved predictions of plant leaf composition.

Finally, the positive results obtained in this study, indicate that it may also be feasible to use hyperspectral imaging data to phenotype for other plant chemical properties. For example, there is a wide interest in breeding improved accessions of energy crops for dedicated biofuel production (for example, biomass sorghum). One of the greatest challenges is to breed for optimized biomass compositions (in particular lignin content which simultaneously determines standability, resistance to fungi and diseases during growth, and to chemical and microbial pretreatments for biomass conversion) without compromising yield or abiotic or biotic stress tolerance (Rooney et al., 2007; Yuan et al., 2008). The lack of a non-destructive, *in vivo* screening tool that can measure biomass composition rapidly is a perceived bottleneck for the effective utilization of high throughput genomics-assisted breeding efforts in sorghum. Our results suggest that hyperspectral imaging could be an effective tool to fill in that technological gap in plant breeders' toolkit. It is quite possible that hyperspectral imaging can predict the chemical compositions of cell wall (such as lignin, cellulose, hemicellulose, and ash) from both plant leaves and stems.

## Conclusion

In this study, the promise of hyperspectral imaging was demonstrated as a non-destructive, *in vivo* tool to measure plant chemical properties including water content, macronutrients, and micronutrients. Statistics from the prediction models indicated that, among the 13 variables, leaf water content and N can be quantified accurately. Predictions of P, K, Mg, Ca, S, Fe, Mn, Cu, and Zn were somewhat less accurate but still quite satisfactorily. Na and B were the only two variables quantified poorly. While there have been many studies reporting the use of other imaging modules (including RGB, fluorescence, and NIR) for high throughput plant phenotyping, the plant traits being investigated were mainly morphological (size and growth) and physiological (chlorophyll and Photosystem II). To the best of our knowledge, this is the first study of using hyperspectral imaging to probe the nutrient concentrations of living plants *in vivo*. The results suggested the high potential of this technique for plant chemical sensing. Future studies to further test the validity of the technique should include experiments that (1) control the variation of individual nutrients in more clearly defined manners, (2) involve more plant species, genotypes, and developmental stages.

## Author contributions

YG contrived the study. PP and VS collected the data. PP analyzed the data. YG and JS interpreted the results. PP and YG drafted the manuscript. JS significantly edited the manuscript.

### Conflict of interest statement

The authors declare that the research was conducted in the absence of any commercial or financial relationships that could be construed as a potential conflict of interest. The reviewer TV and handling Editor declared their shared affiliation, and the handling Editor states that the process met the standards of a fair and objective review.

## Supplementary material

Supplementary Figure 1 Partial least squares regression models for the selected variables of water content (WC), nitrogen (N), phosphorus (P), calcium (Ca), and zinc (Zn).

Supplementary Figure 2 Pairwise scatter plots and correlation matrix of plant nutrient concentrations. Black dots are maize plants and red dots are soybean plants. Pearson's correlation coefficients are significant at 0.05, 0.01, and 0.001 level with one, two, and three stars.

Supplementary Table 1 Results of Welsh's two sample *T*-test to compare the mean of the calibration and validation sets.

Supplementary Table 2 Cross-validation results of using hyperspectral images to predict plant leaf water content, macronutrients, and micronutrients with partial least squares regression, by constructing models for maize and soybean separately.
