# Supplementary figures and images for: High Throughput In vivo Analysis of Plant Leaf Chemical Properties Using Hyperspectral Imaging
Source: Front Plant Sci. 2017 Aug 3;8:1348. doi: 10.3389/fpls.2017.01348 (PMC5540889; doi:10.3389/fpls.2017.01348)

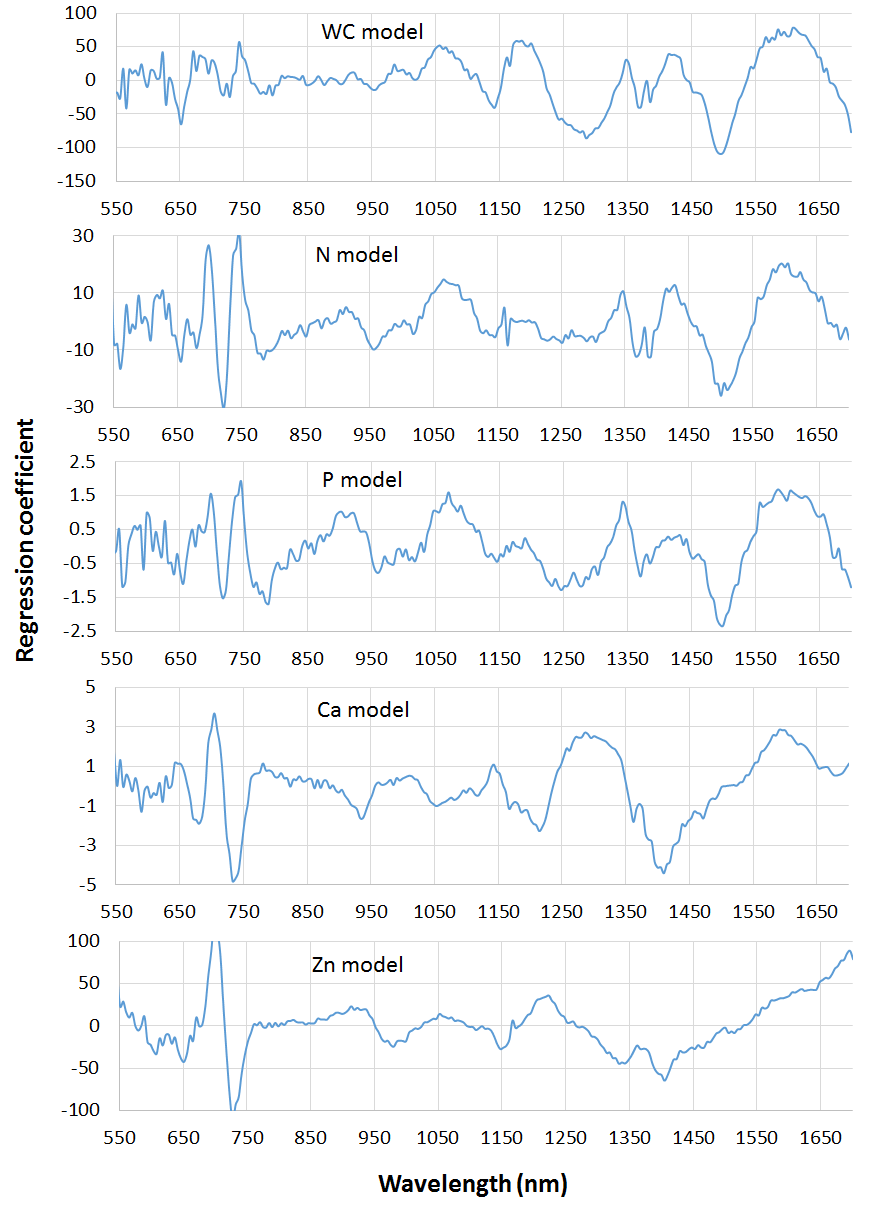

Supplement: Supplementary Figure 1 — Partial least squares regression models for the selected variables of water content (WC), nitrogen (N), phosphorus (P), calcium (Ca), and zinc (Zn). [file Image1.PNG]

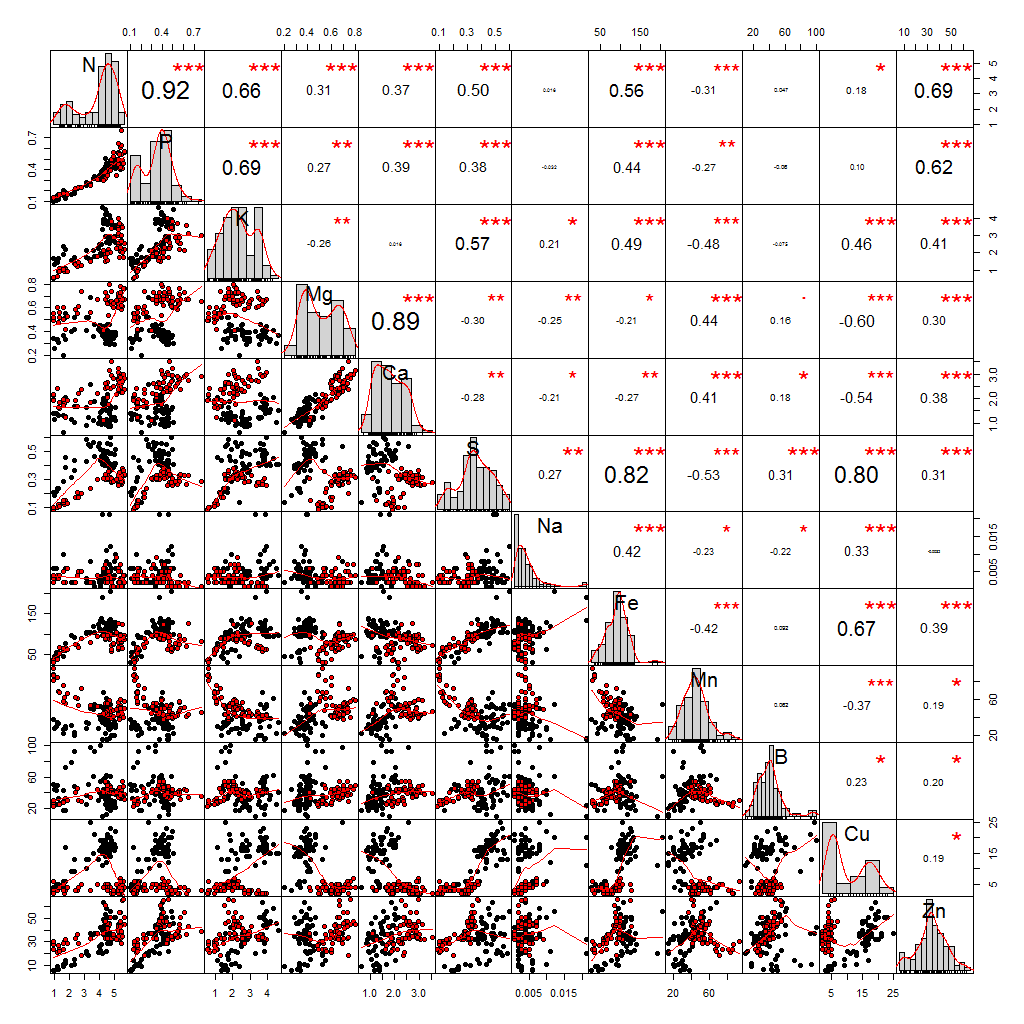

Supplement: Supplementary Figure 2 — Pairwise scatter plots and correlation matrix of plant nutrient concentrations. Black dots are maize plants and red dots are soybean plants. Pearson's correlation coefficients are significant at 0.05, 0.01, and 0.001 level with one, two, and three stars. [file Image2.PNG]
