# Supplementary material for: High Throughput In vivo Analysis of Plant Leaf Chemical Properties Using Hyperspectral Imaging
Source: Front Plant Sci. 2017 Aug 3;8:1348. doi: 10.3389/fpls.2017.01348 (PMC5540889; doi:10.3389/fpls.2017.01348)
Supplement: Supplementary Table 1 — Results of Welsh's two sample T-test to compare the mean of the calibration and validation sets. [file Table1.DOCX]

Supplementary Material

High throughput in vivo analysis of plant leaf chemical properties using hyperspectral imaging

Piyush Pandey, Yufeng Ge*, Vincent Stoeger, James C. Schnable

*** Correspondence:** Yufeng Ge: yge2@unl.edu

## Supplementary Tables

Supplementary Table 1. Results of Welsh’s two sample T test to compare the mean of the calibration and validation sets. The last column gives the p-value of the t-statistic for each variable. No significant difference between the calibration and the validation set was detected for any variable (at 0.05 level), indicating balanced split of data for effective model calibration and validation.

|  | Calibration Set (n=60) | | | Validation Set (n=60) | | |  | p-value |
| --- | --- | --- | --- | --- | --- | --- | --- | --- |
|  | Max. | Mean | Min. | Max. | Mean | Min. |  |  |
| Water Content (%) | 91.0 | 81.0 | 68.2 | 90.4 | 79.8 | 68.5 |  | 0.327 |
|  |  |  |  |  |  |  |  |  |
| N (%) | 5.68 | 4.07 | 0.96 | 5.52 | 3.84 | 0.96 |  | 0.389 |
| P (%) | 0.77 | 0.38 | 0.10 | 0.65 | 0.35 | 0.11 |  | 0.271 |
| K (%) | 4.62 | 2.52 | 0.53 | 4.32 | 2.39 | 0.59 |  | 0.458 |
| Mg (%) | 0.80 | 0.51 | 0.20 | 0.74 | 0.51 | 0.26 |  | 0.869 |
| Ca (%) | 3.21 | 1.88 | 0.63 | 3.53 | 1.93 | 0.85 |  | 0.706 |
| S (%) | 0.60 | 0.35 | 0.09 | 0.57 | 0.34 | 0.10 |  | 0.650 |
|  |  |  |  |  |  |  |  |  |
| Na (%) | 0.021 | 0.0035 | 0.001 | 0.021 | 0.003 | 0.001 |  | 0.863 |
| Fe (ppm) | 205 | 93.7 | 29 | 191 | 89.6 | 30 |  | 0.448 |
| Mn (ppm) | 87 | 43.9 | 15 | 94 | 44.5 | 15 |  | 0.826 |
| B (ppm) | 92 | 38 | 13 | 100 | 43 | 10 |  | 0.079 |
| Cu (ppm) | 23 | 9.3 | 2 | 25 | 9.2 | 2 |  | 0.957 |
| Zn (ppm) | 66 | 34.6 | 6 | 65 | 34.8 | 6 |  | 0.923 |

Supplementary Table 2. Cross-validation results of using hyperspectral images to predict plant leaf water content, macronutrients, and micronutrients with partial least squares regression, by constructing models for Maize and Soybean separately.

|  |  | Maize Set (n = 60) | |  | Soybean Set (n = 60) | |
| --- | --- | --- | --- | --- | --- | --- |
|  |  | R^2^ | RPD |  | R^2^ | RPD |
| Water Content (%) |  | 0.91 | 3.39 |  | 0.91 | 3.35 |
|  |  |  |  |  |  |  |
| N (%) |  | 0.93 | 3.82 |  | 0.94 | 4.10 |
| P (%) |  | 0.88 | 2.88 |  | 0.79 | 2.21 |
| K (%) |  | 0.88 | 2.89 |  | 0.85 | 2.61 |
| Mg (%) |  | 0.64 | 1.75 |  | 0.69 | 1.83 |
| Ca (%) |  | 0.67 | 1.68 |  | 0.70 | 1.80 |
| S (%) |  | 0.71 | 1.87 |  | 0.80 | 2.28 |
|  |  |  |  |  |  |  |
| Na (%) |  | 0.13 | 1.09 |  | 0.16 | 1.11 |
| Fe (ppm) |  | 0.51 | 1.47 |  | 0.77 | 2.14 |
| Mn (ppm) |  | 0.56 | 1.54 |  | 0.80 | 2.26 |
| B (ppm) |  | 0.33 | 1.24 |  | 0.39 | 1.30 |
| Cu (ppm) |  | 0.75 | 1.92 |  | 0.65 | 1.63 |
| Zn (ppm) |  | 0.74 | 1.98 |  | 0.57 | 1.55 |
